# Geotechnical Behavior of Xanthan Gum-Stabilized Clay Reinforced with Polypropylene Fibers

**DOI:** 10.3390/polym17030363

**Published:** 2025-01-28

**Authors:** Jair de Jesús Arrieta Baldovino, Yamid E. Nuñez de la Rosa, Oriana Palma Calabokis, Jesús Alberto Alcalá Vergara, Luis Carlos Suárez López

**Affiliations:** 1 Civil Engineering Program, Universidad de Cartagena, Cartagena de Indias 130015, Colombia; jalcalav@unicartagena.edu.co (J.A.A.V.); lsuarezl@unicartagena.edu.co (L.C.S.L.); 2 Faculty of Engineering and Basic Sciences, Fundación Universitaria Los Libertadores, Bogota 110231, Colombia; opalmac@libertadores.edu.co

**Keywords:** Xanthan Gum, porosity/binder index, strength, stiffness, clay, polypropylene fibers

## Abstract

The use of biopolymers like Xanthan Gum (XG) for soil stabilization offers an eco-friendly alternative, enhancing soil properties while reducing CO_2_ emissions, gaining attention in sustainable engineering. This study investigated the interaction and geotechnical improvements of clay mixed with XG and polypropylene fibers (PPF). Biopolymer was used in proportions of 1%, 3%, and 5%, while the PPF percentage was kept constant at 0.5% by weight. Additionally, the molding density was varied at 1.65 g/cm^3^, 1.70 g/cm^3^, and 1.76 g/cm^3^. A total of 108 specimens were prepared using two curing times (28 and 90 days), and the samples were subjected to unconfined compressive strength (UCS) tests, ultrasonic pulse velocity (UPV), and Scanning Electron Microscopy (SEM). The results demonstrate that the addition of XG and PPF in the specified proportions provides significant mechanical improvements to the stabilized soil. The curing time had a notable impact on these improvements; a curing time of 90 days resulted in strength increases of up to 37% compared to 28 days, while the maximum dry density improved this property by up to 87% compared to the minimum density. The incorporation of PPF enhanced strength by 53.93%, while stiffness increased by 63.55%. Additionally, the strength ($q_{u}$) and stiffness ($G_{o}$) results were successfully correlated using the porosity/binder index $\eta /B_{iv}$, achieving determination coefficients (R²) greater than 0.90 and 0.80, respectively.

## 1. Introduction

Soil stabilization is a fundamental technique to improve the mechanical properties of problematic soils, such as low-plasticity clays. These soils exhibit high compressibility, low strength, and significant susceptibility to wetting and drying cycles, posing challenges for the construction of durable and safe infrastructure [1,2]. Traditionally, materials such as Portland cement and lime have been employed due to their effectiveness. However, their use is associated with significant environmental impacts, including the emission of greenhouse gases. Several studies highlight that traditional cementitious products and some synthetic materials produce large quantities of CO_2_ during their production [3,4,5,6]. Specifically, each ton of cement generates approximately one ton of CO_2_, while lime production emits around 0.86 tons of CO_2_ per ton produced [7,8,9]. These emissions account for approximately 8% of global CO_2_ emissions, significantly contributing to climate change [10,11].

In addition to environmental impacts, the use of these stabilizers can alter groundwater pH, negatively affecting local ecosystems and posing additional environmental risks [12,13]. These limitations have driven the search for sustainable alternatives, increasing interest in unconventional stabilizers, such as polymers, resins, acids, silicates, enzymes, and waste materials, including fly ash [14,15,16,17]. Polymers, in particular, help form physicochemical bonds between soil minerals and reduce porosity within the soil matrix [18,19]. Biopolymers, on the other hand, stand out as natural, carbon-neutral, and energy-efficient alternatives, suitable for various geotechnical applications [20].

Among the biopolymers most studied for addressing geotechnical problems are Guar Gum (GG), Microcrystalline Cellulose (MCC) [20], Chitosan, Persian Gum [21], β-Glucan (BG), and γ-Polyglutamic Acid (GPA) [22], and Zein Biopolymer [23]. However, XG has gained particular attention in geotechnical engineering due to the recent reduction in its market price and its effectiveness in mitigating soil weaknesses [24]. This extracellular polysaccharide, secreted by *Xanthomonas campestris* [25,26], forms cohesive bonds, improves mechanical strength, and increases soil cohesion even at low concentrations ranging from 1% to 2% by weight [1,2,11,19,25,27,28,29]. Studies have demonstrated that XG can triple soil cohesion, reduce susceptibility to wetting-drying cycles, and significantly enhance durability [30,31]. Additionally, Moghal et al. [13] highlight the potential of biopolymers such as XG and GG to stabilize various soil types, including mine tailings.

The reinforcement of soils with randomly distributed fibers is another effective technique that enhances soil engineering properties. Unlike traditional geosynthetics, fibers, such as Polypropylene Fibers (PPF), are homogeneously mixed with the soil, avoiding weak planes parallel to the reinforcement [32]. These fibers increase the unconfined compressive strength (UCS) and reduce both compressibility and swelling of the soil. For instance, the addition of 1% PPF can reduce the compression index by 69% and the swelling index by 78%, while also improving soil cohesion by forming a stable structural network [33,34].

Singh et al. [2] evaluated the effect of XG on expansive soils using proportions of 0%, 0.2%, 0.5%, 0.8%, and 1.0% by weight. The results indicate that the plasticity index of the soil initially increases with the addition of XG but significantly decreases beyond 0.5%. Although compressibility shows a slight increase, swelling pressure, free swell, and hydraulic conductivity decrease notably, while compressive strength and durability improve. Similarly, Khalid et al. [33] investigated the use of PPF in proportions ranging from 0.5% to 1.5% in clayey soils, observing increases in liquid and plastic limits, as well as in UCS. Adding 1% PPF significantly reduced soil compressibility, lowering the compression index by 69% and the swelling index by 78%.

Baldovino et al. [31] analyzed the change from the porosity/Binder ratio to the porosity/Xanthan gum ratio ($\eta /B_{iv}$). This parameter relates the porosity of the compacted mixture (η) to the volumetric content of XG ($B_{iv}$), adjusted by an internal exponent (x), similar to the interaction between cement and clay in stabilized mixtures [34]. They found that this parameter adjusted for XG is 0.04, compared to 0.28 for Portland cement. This suggests that porosity has a more significant impact on the strength development of XG-treated soils. To counterbalance this effect, increasing the XG content in the mixtures is necessary to offset the influence of voids in the material.

Recent studies [35,36,37] highlight the effectiveness of XG in the stabilization of different soils. Missoum et al. [35] found that the biopolymer improves the shear strength and cohesion of sandy soils and also incorporated fine particles as an addition, observing an amplification of the improvements. On the other hand, Chang et al. [36] emphasized that the interaction between XG and soil is influenced by pore fluid chemistry, where XG can reduce the liquid limit through particle aggregation or increase it via hydrogel formation. Studying the addition of XG to clays, Barani and Barfar [37] concluded that XG-treated clays exhibit higher fracture energy and durability, particularly in dry states, addressing challenges associated with high water content.

The combination of XG and PPF represents a synergistic and innovative approach to soil stabilization. While XG enhances cohesion and fills soil voids, fibers mechanically reinforce the structure, creating a dense and resilient matrix. Scanning electron microscopy (SEM) analyses confirm that this combination reduces porosity and improves interconnection between particles and fibers, resulting in a more cohesive and stable material [1,30]. Samples treated with this mixture have shown greater strength and durability, remaining intact even after extended periods of water immersion, unlike untreated soils, which rapidly disintegrate [2,30].

The present study aims to analyze the mechanical behavior of a clay (CL) from the northern region of Cartagena de Indias (Colombia) [38], stabilized with XG and to evaluate the effect of adding PPF as reinforcement. The goal is to reduce the required percentage of XG to achieve specific values of strength ($q_{u}$) and stiffness ${(G}_{o})$, optimizing the stabilization process. The mixtures will be subjected to curing times of 28 and 90 days and evaluated through unconfined compression tests to determine strength properties and ultrasonic pulse measurements to analyze stiffness. This analysis will be complemented by scanning electron microscopy with energy-dispersive spectroscopy (SEM-EDS), along with the development of predictive equations based on the porosity/binder index ($\eta /B_{iv}$). Together, this comprehensive approach will provide insights into both the mechanical and microstructural characteristics of the stabilized mixtures, promoting a more efficient and sustainable solution for improving clay soils.

The limited research addressing the interaction of these materials (i.e., XG and PPF), particularly in clays, combined with the application of the porosity-binder index capable of generating equations and dosage curves from experimental data, represents a significant advancement in soil stabilization through sustainable alternatives.

## 2. Materials and Methods

This study aims to experimentally evaluate the improvement of a low-plasticity clay (CL) soil through the incorporation of Xanthan Gum (XG) and Polypropylene Fibers (PPF). Mixtures were prepared with XG dosages of 1%, 3%, and 5%, relative to the dry mass of the soil, along with PPF at 0.5% of the total sample mass. A total of 108 specimens were produced, subjected to curing periods of 28 and 90 days, with variations in dry density. The specimens were assessed through unconfined compressive strength (UCS) tests and ultrasonic pulse velocity (UPV).

### 2.1. Materials

**Figure 1:**
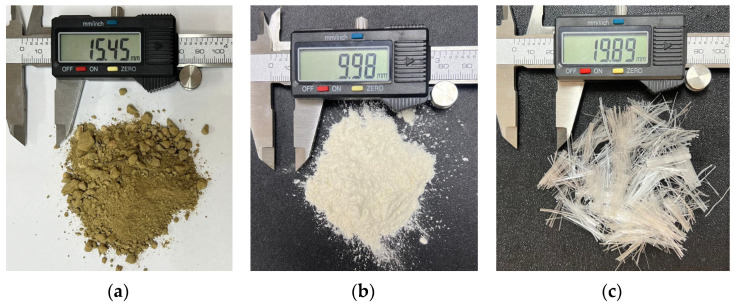
Materials used in the research: (**a**) Clay (CL), (**b**) Biopolymer XG, (**c**) PPF.

A Clay, XG, and PPF are the raw materials used in this research (Figure 1). Acuña et al. [38] previously investigated the dispersivity level of this clay using the pinhole test and the crumb test. Additionally, Roman et al. [39] analyzed the mechanical behavior of this soil, including its strength and stiffness, along with its microstructure. This analysis was conducted using crushed limestone waste as an improvement material, evaluating the soil’s properties through the porosity-binder index.

**Figure 2:**
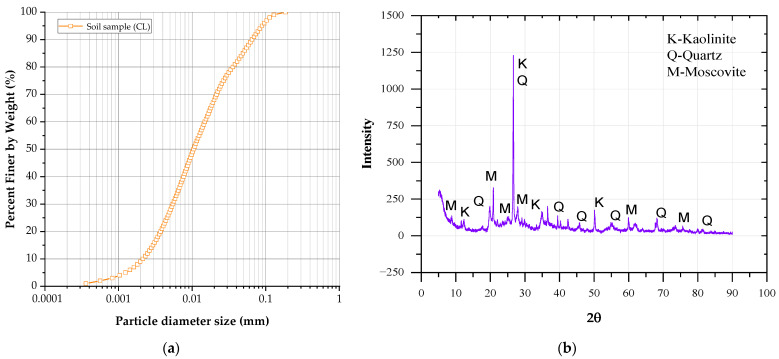
(**a**) The granulometric curve of soil sample. (**b**) X-ray diffraction (XRD) of the soil sample.

Figure 2 shows the particle size distribution of the soil and its mineralogical composition.

Table 1 provides the soil’s chemical composition obtained through X-ray fluorescence. The soil was composed mainly of kaolinite clay minerals.

Table 2 presents its geotechnical characterization. The soil was classified as low-plasticity clay (CL) according to the Unified Soil Classification System (USCS) [40]. Its specific gravity was measured as 2.80 following ASTM D854 [41]. The particle size distribution revealed 10% clay, 78% silt, and 12% sand, with coefficients of curvature and uniformity of 0.96 and 7.14, respectively. The additional morphological composition of the sample and details of the soil’s geotechnical properties can be found in Roman et al. [39].

The XG used in this study is a polysaccharide produced by the bacterium *Xanthomonas campestris*. Its structure consists of repeating pentasaccharide units containing glucose, mannose, and glucuronic acid in a molar ratio of 2:2:1 [26,31,46]. The density of XG (ρXG) was found to be 1.5 g/cm^3^, with a pH of 6.34 and a viscosity of 1200 MPa∙s.

**Figure 3:**
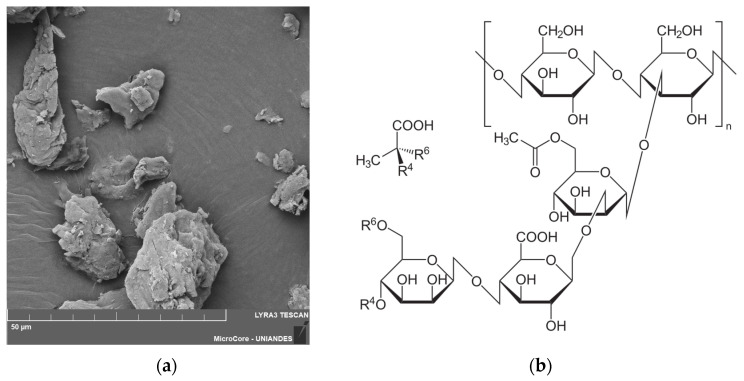
(**a**) XG morphology recorded in the SEM analysis (**b**) Molecular structure of XG.

XG has an elemental composition primarily consisting of carbon (54.05%), oxygen (43.47%), sodium (1.59%), and calcium (0.89%) (Figure 3). This chemical composition enhances its ability to form ionic and covalent bonds, improving cohesion and the structural integrity of the treated soil.

PPF were sourced from a local supplier. These fibers have a length of 20 mm and a density (ρPPF) of 0.91 g/cm^3^.

### 2.2. Methodology

#### 2.2.1. Specimen Molding and Preparation

**Figure 4:**
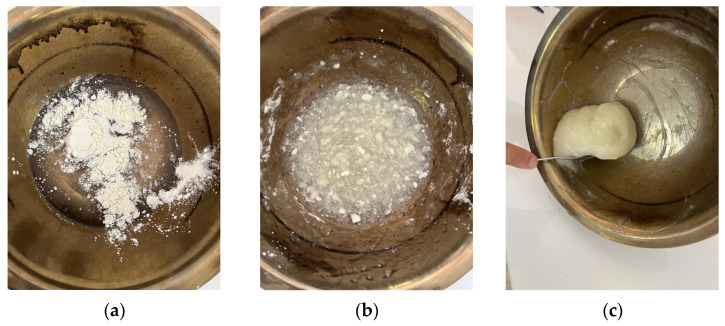
Manual mixing of XG in water. (**a**) Addition of XG in water (**b**) Partial mixing of the gum in water. (**c**) Final homogeneous mixture of XG.

XG was mixed using distilled water for dissolution with soil in proportions of 1%, 3%, and 5% these percentages were selected based on the literature [29,31,46,47]. The water content was adjusted based on the maximum dry unit weight, and the mixture was manually prepared to ensure homogeneity (Figure 4).

A total of 108 specimens were fabricated, following the distribution outlined in Table 3. These samples were made in cylindrical metal molds with a diameter of 2 inches and a height of 4 inches, complying with the 1:2 ratio specified by ASTM D1632 [48].

Each specimen was compacted in three layers, with the surface of each layer being scarified to ensure uniformity in density and overall characteristics. Additionally, the same number of specimens was prepared with the inclusion of 0.5% PPF relative to the dry weight of the soil. All specimens were compacted in triplicate to ensure consistency in the results.

Specific acceptance and rejection criteria for the specimens were applied during the process, based on recommendations from the literature [49,50]. The specimens underwent a curing process under controlled temperature and humidity conditions to prevent moisture loss, with curing periods of 28 and 90 days. Additional curing conditions and controls were implemented following the methodology described by Baldovino et al. [31].

#### 2.2.2. Program of Ultrasonic Pulse Velocity Test and UCS Test

Upon completion of the curing process, the specimens were removed from the humid chamber and immersed in distilled water for 24 h to reduce matric suction, as suggested by previous studies [31,39,49,50,51].

To determine the stiffness of the samples, the Pundit Lab Plus equipment (Proceq, Schwerzenbach, Switzerland) was used following the ASTM C597 standard [52]. This procedure provided the necessary values to calculate the small-strain shear modulus ($G_{o}$). Compression waves were generated using transducers at a frequency of 54 kHz, while shear waves were recorded at 250 kHz.

Subsequently, UCS tests were conducted in accordance with the ASTM D2166 standard [53]. For this purpose, the specimens were subjected to axial loading using a 50 kN multitest hydraulic press with a sensitivity of 0.1 kN and a controlled deformation rate of 1.15 mm/min.

#### 2.2.3. Microstructural Analysis

SEM analyses were performed on strategically selected points of the samples, reaching magnifications of up to 5000×. The resolution was adjusted based on the material: up to 5 microns for Soil-XG samples and 100 microns for Soil-XG-PPF samples. These analyses were conducted using the LYRA-3 dual-beam SEM-FIB system manufactured by TESCAN (Tescan Orsay Holding, Brno-Kohoutovice, Czech Republic).

#### 2.2.4. Application of the Porosity-Binder Index

The porosity/binder index ($\eta /B_{iv}$), introduced by Consoli et al. [54], has become a widely used tool for analyzing the UCS of artificially cemented soils [31,34,39,49,51,54,55,56]. Volumetric Binder content ($B_{iv}$) is calculated using the original formula proposed by Baldovino et al. [57]. The porosity (η) adapted to account for the addition of XG and PPF (Equation (1)).

(1) $$\eta =100-\frac{100\cdot \gamma_{d}\cdot V_{s}}{V_{specimen}}\cdot \left[\frac{1}{G_{s}\cdot \left(1+\frac{{XG}_{\%}+{PPF}_{\%}}{100}\right)}+\frac{{XG}_{\%}}{100\cdot \rho XG\cdot \left(1+\frac{{XG}_{\%}+{PPF}_{\%}}{100}\right)}+\frac{{PPF}_{\%}}{100\cdot \rho PPF\cdot \left(1+\frac{2\cdot {PPF}_{\%}}{100}\right)}\right]$$

The Equation (1) incorporates the percentage of XG $\left({XG}_{\%}\right)$ and PPF (${PPF}_{\%}$), the material densities defined in Section 2.1, the specific gravity of the soil ($G_{s}$) presented in Table 1, the molding densities ($\gamma_{d}$) indicated in Table 3, as well as the volume of the specimen ($V_{specimen}$) and the soil volume ($V_{s}$).

The UCS $(q_{u}$) and the initial stiffness at small strains ($G_{o}$) are related to the *η*/*B_iv_* index through adjustments based on two empirical exponents (*x* and *C*) and an empirical constant *A_q_*. These properties are expressed in kPa and MPa, respectively, as defined in Equation (2).

(2) $$q_{u}\;V\;G_{o}=A_{q}{\left[\frac{\eta }{{\left(B_{iv}\right)}^{x}}\right]}^{-C}\;$$

The scalar *A_q_* is defined by the interplay between the strength of cementation bonds and the soil matrix properties, representing a key parameter in modeling resistance [58]. This value is also influenced by factors related to the physical and chemical characteristics of the soil, enabling a more accurate representation of structural behavior under varying conditions [59].

## 3. Results and Discussion

The results obtained in the improvement of a clayey soil using XG as a stabilizing agent are presented and analyzed below. This study evaluated key factors such as dry unit weight, curing time, and binder content, considering their influence on the UCS ($q_{u}$) and UPV ($G_{o}$) of the resulting mixtures.

Additionally, the mechanical and microstructural behavior induced by the incorporation of PPF was examined, identifying structural changes and their impact on the properties of the treated material. Furthermore, the application of a rational predictive methodology based on the porosity/binder index, proposed by Consoli et al. [54] was validated for predicting the stiffness and strength results.

### 3.1. Influence of XG Content, Dry Unit Weight and Curing Time on the Strength and Stiffnes of a Stabilized Clayey Soil

**Figure 5:**
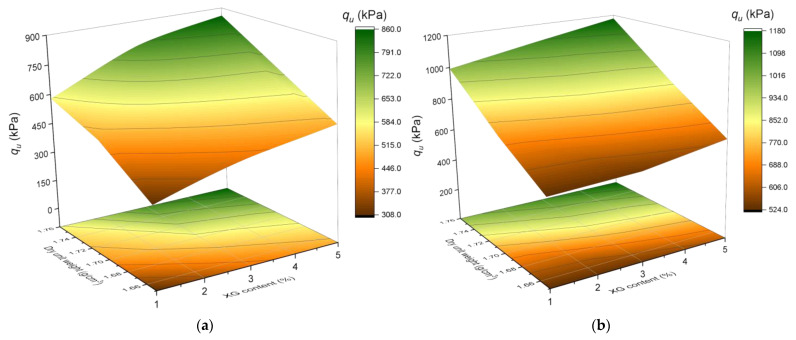
Influence of curing time, dry unit weight (g/cm^3^), and XG content (%) on compressive strength. (**a**) Compressive strength results after 28 days of curing. (**b**) Compressive strength results after 90 days of curing.

**Figure 6:**
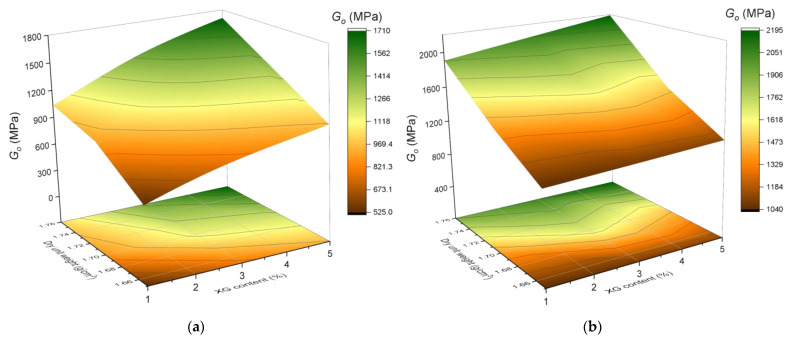
Influence of curing time, dry unit weight (g/cm^3^), and XG content (%) on stiffness. (**a**) Stiffness results after 28 days of curing. (**b**) Stiffness results after 90 days of curing.

Figure 5 and Figure 6 demonstrate that curing time, XG content, and dry density are key factors that significantly affect both the UCS and stiffness of the stabilized soil. Samples cured for 90 days show notable improvements compared to those cured for 28 days, with increases of up to 37% in UCS and even greater values for stiffness. The addition of XG to the soil contributes to the retention of water content within the soil matrix pores, which can enhance soil cohesion. This, in turn, influences the expansion and contraction of the soil due to moisture changes, improving both stability and strength [31].

Increasing the XG content from 1% to 5% leads to significant improvements in both properties. In terms of UCS, the increases range between 30% and 39%, depending on the initial soil conditions, while stiffness exhibits proportional increments under the same conditions. Additionally, increasing the molding density from 1.65 g/cm^3^ to 1.76 g/cm^3^ results in UCS improvements of up to 87%, while stiffness also shows significant enhancements, reaching more than double the initial values in some cases. These findings highlight the importance of adequate initial compaction to maximize the positive impact of the stabilizer on the interaction between soil particles.

Moreover, in Figure 5b and Figure 6b, noticeable changes in the trends of UCS and stiffness lines can be observed compared to Figure 5a and Figure 6a. After transitioning to a curing time of 90 days, these lines gain greater steepness relative to the dry unit weight axis, suggesting that this parameter may have a more pronounced influence as curing times increase.

### 3.2. Influence of XG Content, Dry Unit Weight, Curing Time, and PPF on the Strength and Stiffnes of a Stabilized Clayey Soil

**Figure 7:**
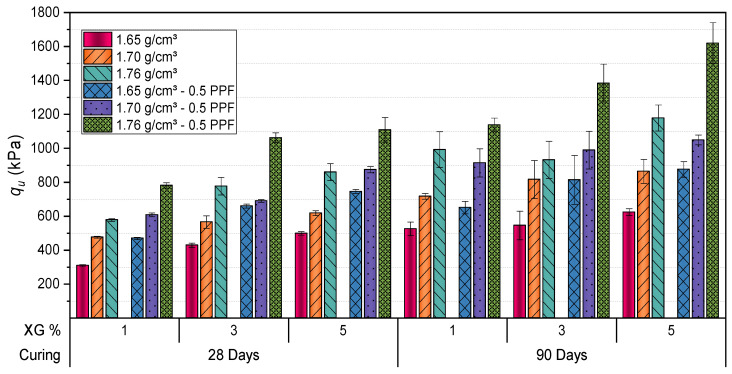
Influence of curing time, dry unit weight (g/cm^3^), XG, and PPF on strength of stabilized Clayey Soil.

**Figure 8:**
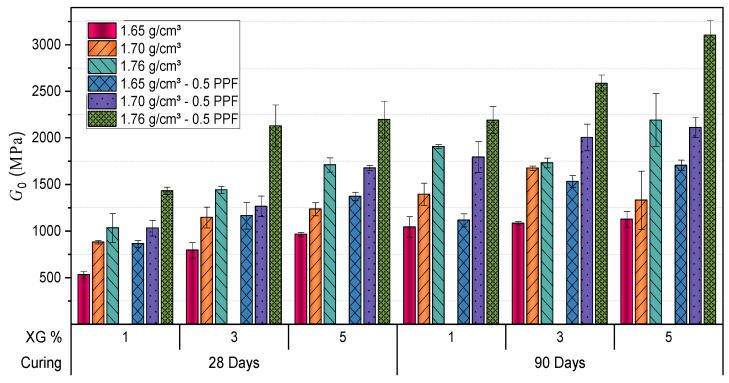
Influence of curing time, dry unit weight (g/cm^3^), XG, and PPF on stiffness of stabilized Clayey Soil.

Figure 7 and Figure 8 show how the addition of PPF significantly impacts the mechanical properties of XG stabilized soil, regardless of the dry unit weight being analyzed. For instance, in mixtures with 1% XG, the data indicate that after 28 days of curing, UCS increased from 308.12 kPa without PPF to 463.86 kPa with 0.5% fibers at a dry unit weight of 1.65 g/cm^3^, representing a 51% increase. When the dry unit weight is increased to 1.76 g/cm^3^, the differences remain evident, reaching a compressive strength of 774.05 kPa with PPF compared to 576.01 kPa without fibers. This 34% increase suggests higher initial density conditions of the mixture.

With 3% XG, significant improvements in soil properties were observed. For samples compacted at a dry unit weight of 1.65 g/cm^3^, the UCS increased from 435.50 kPa (no fibers) to 670.90 kPa (with 0.5% PPF), representing a 54% enhancement. At higher dry unit weights, such as 1.76 g/cm^3^, the strength rose from 770.69 kPa to 1062.38 kPa, reflecting a 38% improvement. Stiffness values exhibited similar trends, increasing from 861.42 MPa to 1071.76 MPa at 1.65 g/cm^3^ and from 1408.44 MPa to 1900.81 MPa at 1.76 g/cm^3^.

These findings align with prior studies demonstrating the ability of XG to enhance interparticle bonding and improve mechanical performance in stabilized soils [27,31].

After 90 days of curing, the effects of stabilization were more pronounced. In samples with 5% XG and a dry unit weight of 1.65 g/cm^3^, UCS increased from 611.50 kPa (no fibers) to 898.57 kPa (with fibers), while stiffness improved from 1162.34 MPa to 1769.10 MPa, reflecting gains of 47% and 52%, respectively. For a dry unit weight of 1.76 g/cm^3^, UCS increased from 1215.50 kPa to 1698.50 kPa, and stiffness rose from 2520.46 MPa to 3247.25 MPa. These results emphasize the importance of compaction and curing time in optimizing the performance of stabilized materials. The progressive formation of cohesive soil-polymer matrices during curing significantly enhances strength and stiffness [60].

Overall, the results confirm the suitability of XG for geotechnical applications. By forming cohesive matrices, XG enhances both the strength and stiffness of treated soils, providing an efficient and sustainable alternative for soil stabilization [27,37]. Proper management of variables such as XG content, compaction, and curing time is crucial to achieving optimal performance in stabilized materials.

**Figure 9:**
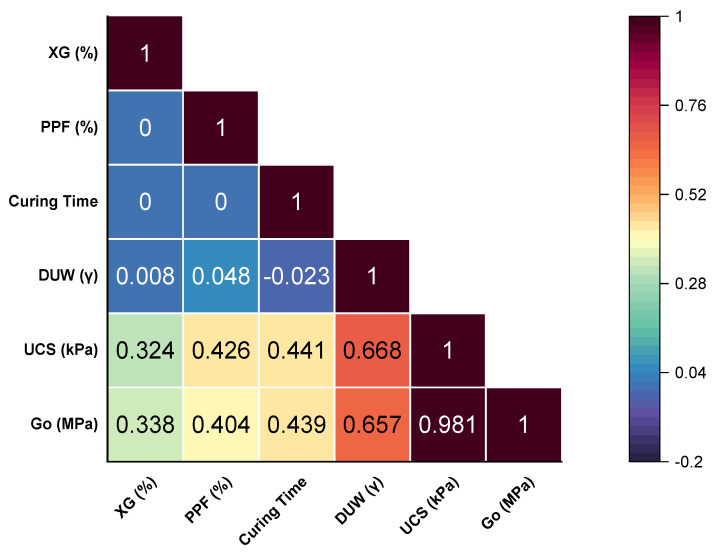
Correlation Matrix of Variables.

The analysis of the mechanical properties of stabilized soils reveals that dry unit weight is the most influential factor on UCS ($q_{u}$) and stiffness ($G_{o}$) (Figure 9). This is reflected in positive correlations of 0.668 and 0.657, respectively, indicating that higher density enhances particle-additive interactions, significantly improving the material’s performance.

Curing time, with correlations of 0.441 and 0.439 for UCS and stiffness, allows chemical reactions to reach their full potential, thereby increasing the structural stability of the soil. Additionally, PPF contribute as reinforcements that distribute stresses, showing a moderate positive impact with values of 0.426 and 0.404.

XG, while exhibiting lower correlations (0.324 for $q_{u}$ and 0.338 for $G_{o}$), acts as a binder that enhances particle cohesion. This highlights its usefulness as a complementary additive in stabilized systems, especially when combined with other factors such as density and curing time.

The combined use of XG and PPF effectively improves the mechanical properties of treated clay soils. Increasing XG content consistently enhances the UCS, as demonstrated in various studies. Weng et al. [61] observed that an optimal XG content of 1.5% to 2% resulted in UCS improvements of up to 2.41 times compared to untreated soils. Although the inclusion levels in the present work were slightly higher, the trends confirm the positive impact of XG as a stabilizer. Similarly, Bozyigit et al. [60] reported that XG increased the dry unit weight of treated soils from 12.75 kN/m^3^ to 14.52 kN/m^3^, while also improving the Atterberg limits. The liquid limit increased by 32% with XG, highlighting its role in altering the plasticity of treated materials.

Extended curing periods further amplify the benefits of XG stabilization. After 90 days, samples prepared at optimum water content demonstrated significant gains in both strength and stiffness. Conversely, excessive water content reduced long-term strength, underscoring the importance of precise preparation conditions. These findings are consistent with previous studies emphasizing the role of curing time in fostering enhanced soil-polymer interactions [16,31].

### 3.3. Application of the Porosity/Binder Index to Predict the Strength and Stiffnes of Compacted Blends

**Figure 10:**
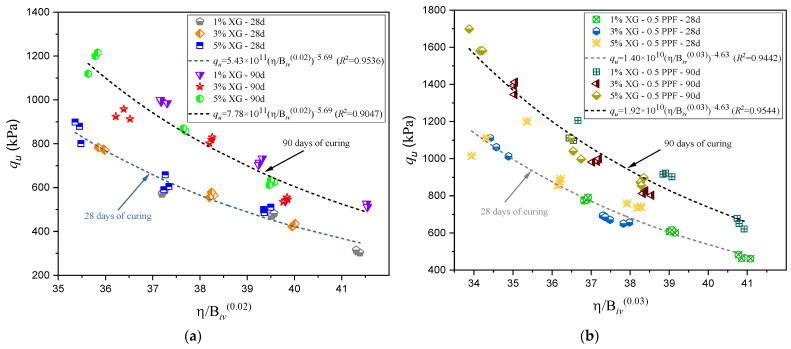
Effect of the Porosity/Binder Index on Predicting UCS. (**a**) Specimens of soil and XG cured for 28 and 90 days. (**b**) Specimens of Soil–XG–PPF tested after 28 and 90 days of curing.

Figure 10a,b depict the Porosity/Binder index adjustments for Soil-XG conditions under two curing times, as well as for the addition of PPF. It was observed that a reduction in porosity led to an increase in the UCS ($q_{u}$) of the prepared mixture, aligning with findings from previous studies [49,50,53,54,55].

The equations derived for UCS ($q_{u}$) incorporate an exponent of 0.02 for the $\eta /B_{iv}$ index in soil-XG mixtures. This value is consistent with the study by Baldovino et al. [31], where the exponent was adjusted to 0.04. The discrepancy may be attributed to the inclusion of two curing periods and the simultaneous adjustment for these conditions.

Conversely, for soil-XG-PPF mixtures, the exponent is adjusted to 0.03, reflecting the mechanical contribution of the fibers, particularly in stress redistribution and structural behavior modification of the stabilized soil.

**Figure 11:**
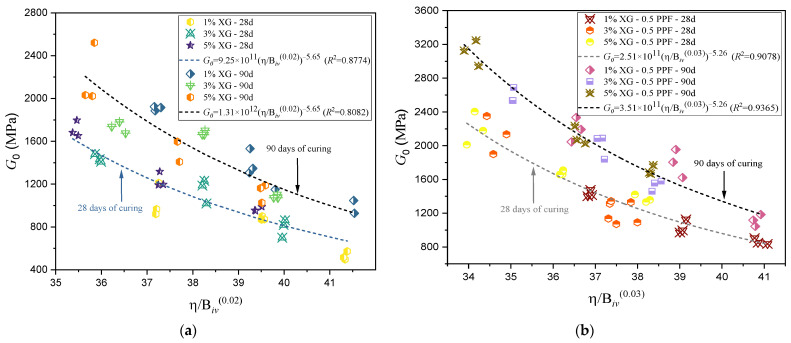
Effect of the Porosity/Binder Index on Predicting Stiffness. (**a**) Specimens of soil and XG cured for 28 and 90 days. (**b**) Specimens of Soil–XG–PPF tested after 28 and 90 days of curing.

An increase in the strength and stiffness of the soil following densification of the soil [62] is possible by applying the appropriate densification method and using suitable proportions of materials in the design. This principle aligns with the findings of the study, where a rational model relating porosity and binder content successfully described the initial stiffness at small deformations for the different soil mixtures evaluated. However, it is important to note that the coefficients of determination for $G_{o}$ showed lower values compared to those obtained for $q_{u}$ (see Table 4 and Table 5). In Figure 11b, the maximum stiffness value was observed for the combination of 5% XG with 0.5% PPF, after 90 days of curing and a density of 1.76 g/cm^3^. Similar to UCS, the stiffness demonstrated a direct relationship with curing time. Figure 11a presents results for specimens without PPF, while Figure 11b includes PPF, confirming that the addition of PPF improves soil stiffness. In cases with equivalent densities, XG percentages, and curing times, higher stiffness values were observed when PPF was incorporated.

### 3.4. Normalization of Equations for the Strength and Stiffness of Compacted Blends

Normalization involves the development of accurate predictive models that eliminate the inherent variations in individual samples, enabling the identification of global and unique trends. In this case, the aim is to derive a suitable equation to estimate $q_{u}$ and $G_{o}$ as a function of the normalized index $\eta /B_{iv}$, with a single potential trend for the studied clay under the various parameters adjusted in the study. In recent studies, Lopez et al. [55] applied normalization to four sands, performing identical tests to evaluate the mechanical properties of the improved soil. To establish a normalized equation, the initial step was determining all normalized strengths and stiffnesses using a specific value of $\eta /{(B}_{iv}^{x}$) = ∇. For the present study, the normalization range of ∇ is between 30 and 45, applicable to both the UCS ($q_{u}$) and the small-strain initial stiffness ($G_{o}$). For $q_{u}$, a value of ∇ = 36 was selected, and for $G_{o}$, ∇ = 34 (see Figure 10 and Figure 11) to calculate the normalized strength *q*_u−n_ and stiffness $G_{o-n}$.

**Figure 12:**
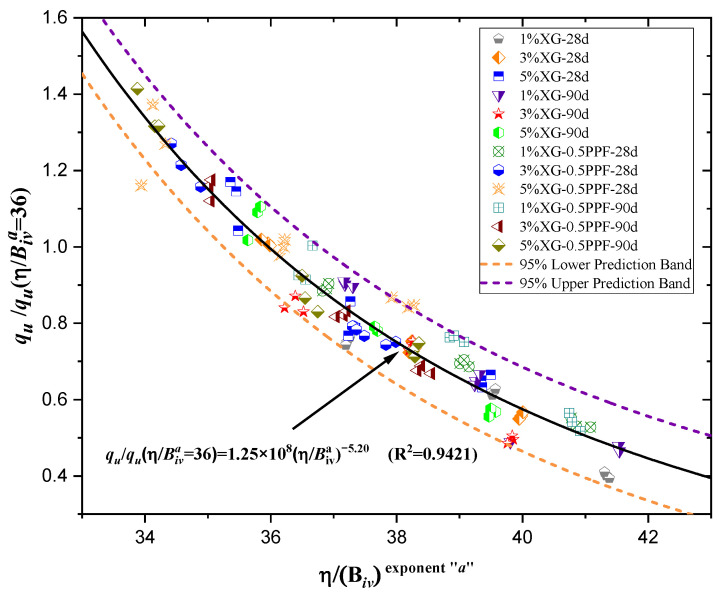
Normalization using the porosity/binder index for UCS ${(q}_{u})$ data.

**Figure 13:**
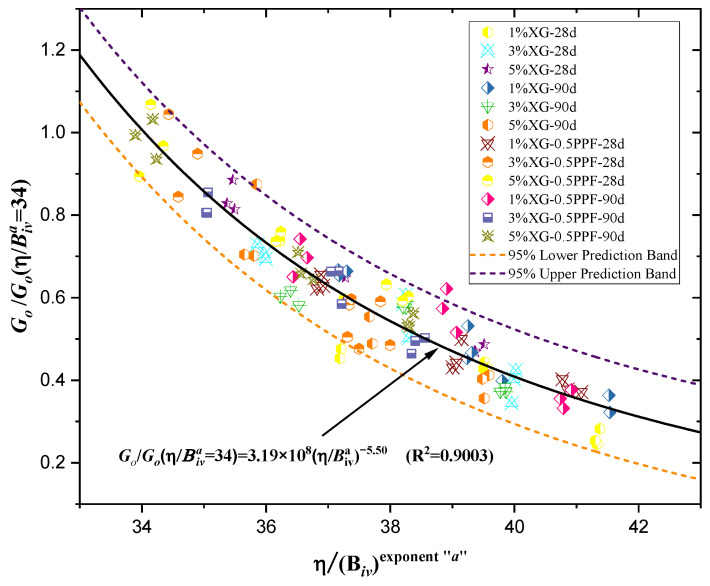
Normalization using the porosity/binder index for stiffness ($G_{o}$) data.

The normalized strengths $q_{u-n}$ are 767.94 kPa for XG-0PPF-28d, 1099.88 kPa for XG-0PPF-90d, 874.79 kPa for XG-0.5PPF-28d, and 1201.24 kPa for XG-0.5PPF-90d. The normalized stiffnesses $G_{o-n}$ are 2029.64 MPa for XG-0PPF-28d, 2882.24 MPa for XG-0PPF-90d, 2251.18 MPa for XG-0.5PPF-28d, and 3145.23 MPa for XG-0.5PPF-90d. After calculating the normalized strength and stiffness values, the UCS and the small-strain initial stiffness for each sample were divided by their corresponding normalized values for the type of sand studied (for both $q_{u}$ and $G_{o}$). This normalization was performed for a total of 108 results of $q_{u}$ and $G_{o}$. The strength and stiffness values used for normalization are provided in Table 6. Figure 12 and Figure 13, and Equation (3) represent the best fit for the normalized strength values based on the chosen parameter, with a normalization coefficient of 0.9421, indicating a unique trend encompassing all experimental and normalized data points.

Similarly, the small-strain initial stiffness data ($G_{o}$) were used to generate Figure 12 and Equation (4), which achieved a determination coefficient of 0.9003. Although this is slightly lower than the fit obtained for the UCS, in both cases, the fit exceeded 0.90. Therefore, it is critical to validate the use of Equations (3) and (4) to describe the mechanical behavior of the studied clay when mixed with varying percentages of XG and PPF.

(3) $$\frac{q_{u}}{q_{u-n}\left(\eta /{\left(B_{iv}\right)}^{a}=36\right)}=1.25\times 10^{8}{\left(\eta /{\left(B_{iv}\right)}^{a}\right)}^{-5.20}\;\left(R^{2}=0.9421\right)$$

(4) $$\frac{G_{o}}{G_{o-n}\left(\eta /{\left(B_{iv}\right)}^{a}=34\right)}=3.19\times 10^{8}{\left(\eta /{\left(B_{iv}\right)}^{a}\right)}^{-5.50}\;\left(R^{2}=0.9003\right)\;$$

### 3.5. Microstructure and Microanalysis of Soil Mixtures

Microstructural analyses of soil mixtures with XG and PPF using SEM-EDS imaging were conducted on a single type of specimen. These specimens were molded with an XG content of 5% and a PPF content of 0.5%, a selection based on the highest mechanical performance values recorded for the soil mixture after a curing period of 90 days.

Biopolymers function as a surface layer that encapsulates soil particles, establishing connections and direct chemical interactions that bind previously separated particles. The behavior of the soil matrix treated with biopolymers is complex and influenced by several factors, including the electrical charges of the biopolymer itself, the inherent cations within the clay, and the total charge density in the system [63].

**Figure 14:**
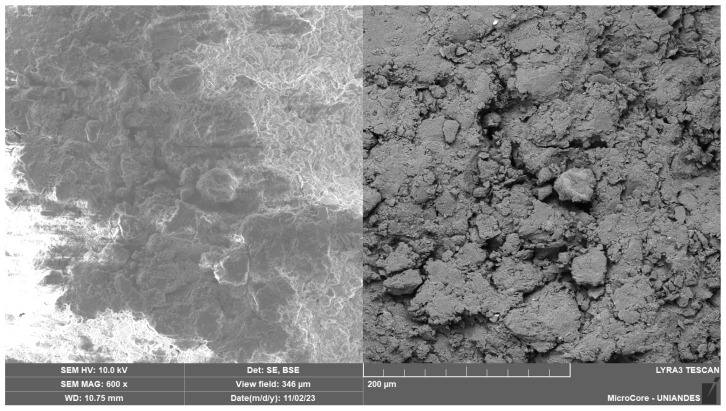
SEM images of soil treated with XG biopolymer.

Figure 14 shows the interface between the soil and XG alone. A homogeneous matrix with reduced voids (low porosity) is observed, as evidenced by a 200 and 20 μm zoom view.

**Figure 15:**
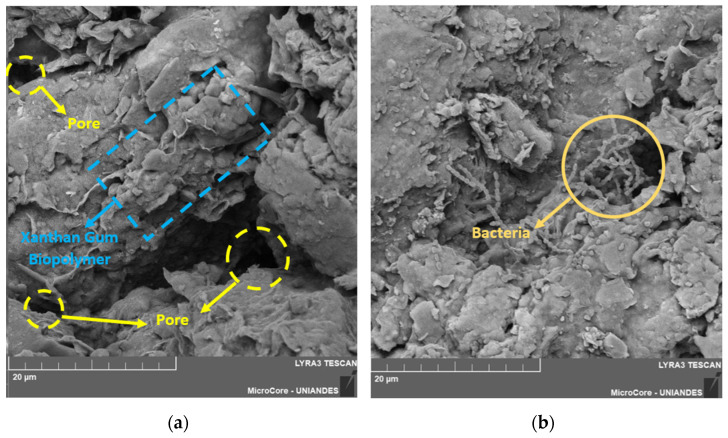
SEM images of soil treated with XG biopolymer: (**a**) Voids at the interface between the soil and the XG. (**b**) *Xanthomonas campestris* bacteria present in the soil-XG matrix.

This confirms why the combination of 5% XG with 90 days of curing exhibited high results in UCS and small-strain initial stiffness. In Figure 15b, certain voids are visible at the soil mixture interface. The reaction between the XG biopolymer and water generates a substantial amount of biopolymer film accumulating on soil surfaces. This biopolymer film, known as hydrogel, provided a denser structure and thicker adhesive bonds between soil particles for the utilized 5% XG content. Similar results were reported in previous studies by Yang et al. [64], which investigated mechanisms for improving biopolymer-reinforced soil stabilized with XG. Lastly, Figure 15b identifies the presence of a dead bacterium, *Xanthomonas campestris*, which is used in the production of XG, an organic-origin biopolymer.

**Figure 16:**
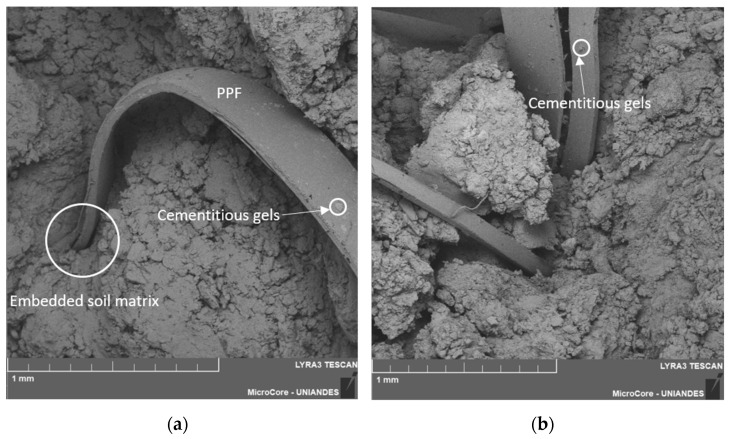
SEM images of Soil–XG–PPF mixture. (**a**) the XG biopolymer content of 5% and 0.5% PPF. (**a**) Interaction between Soil-XG-PPF. (**b**) Reinforcement matrix between Soil-XG-PPF.

Figure 16a,b demonstrate that the combination of PPF and soil-XG led to the formation of a reinforcement matrix, further emphasizing the significance of low porosity. The lighter-colored zones indicate the presence of XG, and in some regions, the PPF adhered to the cementitious gels, enhancing the roughness of the fibers. Additionally, a more solid matrix was evident due to the interaction between the soil and XG. This microscopic analysis corroborates the superior mechanical performance observed for the soil mixture containing 0.5% PPF and 5% XG. Lang et al. [65] reported similar findings in their microanalysis of a sedimented soil matrix stabilized with cement and PPF. Their study revealed that hydration products filled microcracks and pores between fibers and soil particles, resulting in a more compact microstructure.

## 4. Conclusions

In this study, the effects of XG and PPF on the geotechnical improvements of clay were evaluated using UCS tests, UPV and SEM-EDS. Based on the results and analyses obtained, the following conclusions can be drawn:

- XG is effective for soil stabilization due to its ability to improve cohesion and moisture retention in treated soils. However, its limited availability and relatively high cost make its use at lower percentages a more sustainable alternative. The combination with PPF presents itself as an alternative, providing adequate strength and stiffness while optimizing resources and costs.
- The results show that extending the curing time from 28 to 90 days increases strength by up to 37%, while raising the dry density from 1.65 to 1.76 g/cm^3^ improves this property by up to 87%. Additionally, the impact of fiber usage is highly significant; regarding strength $\left(q_{u}\right)$, the minimum and maximum percentage increases observed between samples without fibers and those with fibers were 14.70% and 53.93%, respectively. For stiffness ($G_{o}$), the minimum and maximum percentage increases were 7.06% and 63.55%, respectively. These factors are crucial for achieving optimal mechanical performance of the soil.
- The porosity/binder index $\left(\eta /B_{iv}\right)$, accurately models the mechanical behavior of soils stabilized with XG, even with the addition of fibers, achieving equations with reliability coefficients R^2^ above 0.9 for strength $\left(q_{u}\right)$ and over 0.8 for stiffness $\left(G_{o}\right)$.
- The normalization of two equations, one for $q_{u}$ and another for $G_{o}$, enabled the derivation of expressions that adapt to all conditions and variations in the XG and PPF percentages used in the samples. These generated determination coefficients (R^2^) of 0.9421 for UCS and 0.9003 for stiffness.
- In the microstructure of the soil mixture incorporating XG and PPF, a denser matrix with reduced porosity was observed due to hydrogel formation. This facilitated a more compact interface, where the fibers acted as reinforcement material, effectively distributing internal stresses and preventing brittle failures.

The future scope of this research lies in optimizing soil-polymer mixtures for various soil types, exploring the development of new biopolymers and sustainable additives, and analyzing the environmental impact associated with their use. All of this aims to generate sufficient information to support and establish regulations, standards, and the validity of biopolymer applications in the field of engineering.

## Figures and Tables

**Table 1:** Chemical composition of soil sample (% by weight).

Materials	SiO_2_	Al_2_O_3_	SO_3_	K_2_O	CaO	Fe_2_O_3_	TiO_2_	LOI
Soil (CL)	66	21.1	4	3.1	3	0.9	0.3	1.6

**Table 2:** Geotechnical properties of the soil sample.

Property of Soil	Standard/Reference	Unit	Value
The specific gravity $\left(G_{s}\right)$	[41]	-	2.8
Plasticity limit, P.L.	[42]	%	26.05
Plastic index, P.I.	[42]	%	15.95
Fine Sand (0.075–0.425 mm)	[40,43]	%	12
Silt (0.002–0.075 mm)	[40,43]	%	78
Clay (<0.002 mm)	[40,43]	%	10
Mean Diameter (*d*_50_)	[40,43]	mm	0.011
Effective Diameter (*d*_10_)	[40,43]	mm	0.0021
Uniformity Coefficient *C*_u_	[40,43]	-	7.14
Coefficient of Curvature *C*_c_	[40,43]	-	0.96
Activity of Clay, A [A = PI/(% < 0.002 mm)]	[44]	-	1.60
USCS Classification	[40]	-	CL
Optimum Moisture Content	[45]	%	18.20
Maximum Dry Unit Weight $\left(\gamma_{smax}\right)$	[45]	g/cm^3^	1.76

**Table 3:** Mixed proportion design for compacted blends of soil, XG, and PPF.

Mix	Weight (%)			Curing Times (d)	Molding γ_d_ (g/cm^3^)	Number of Specimens
	Soil	XG	PPF			
Soil–XG	100	1	-	28, 90	1.65, 1.70, 1.76	18
	100	3	-	28, 90	1.65, 1.70, 1.76	18
	100	5	-	28, 90	1.65, 1.70, 1.76	18
Soil–XG–PPF	100	1	0.5	28, 90	1.65, 1.70, 1.76	18
	100	3	0.5	28, 90	1.65, 1.70, 1.76	18
	100	5	0.5	28, 90	1.65, 1.70, 1.76	18

**Table 4:** UCS $\left(q_{u}\right)$ equation of compacted blends.

Type of Mix	Compressive Strength Equation	Coefficient R^2^
Soil–XG 28d	$q_{u}=5.43\times 10^{11}{\left(\eta /{\left(B_{iv}\right)}^{0.02}\right)}^{-5.69}$	0.9536
Soil–XG 90d	$q_{u}=7.78\times 10^{11}{\left(\eta /{\left(B_{iv}\right)}^{0.02}\right)}^{-5.69}$	0.9047
Soil–XG–PPF 28d	$q_{u}=1.40\times 10^{10}{\left(\eta /{\left(B_{iv}\right)}^{0.03}\right)}^{-4.63}$	0.9442
Soil–XG–PPF 90d	$q_{u}=1.93\times 10^{10}{\left(\eta /{\left(B_{iv}\right)}^{0.03}\right)}^{-4.63}$	0.9544

**Table 5:** Stiffness Equation of compacted blends.

**Type of Mix**	**Stiffness Equation**	**Coefficient R^2^**
Soil–XG 28d	$G_{o}=9.25\times 10^{11}{\left(\eta /{\left(B_{iv}\right)}^{0.02}\right)}^{-5.65}$	0.8774
Soil–XG 90d	$G_{o}=1.31\times 10^{12}{\left(\eta /{\left(B_{iv}\right)}^{0.02}\right)}^{-5.65}$	0.8082
Soil–XG–PPF 28d	$G_{o}=2.51\times 10^{11}{\left(\eta /{\left(B_{iv}\right)}^{0.03}\right)}^{-5.26}$	0.9078
Soil–XG–PPF 90d	$G_{o}=3.51\times 10^{11}{\left(\eta /{\left(B_{iv}\right)}^{0.03}\right)}^{-4.63}$	0.9365

**Table 6:** Normalization data.

Mix	Normalization Index (∇)		For Normalization	
	$q_{u-n}$	$G_{o-n}$	$q_{u}(kPa)$	$G_{o}(MPa)$
Soil–XG 28d	$\eta /{\left(B_{iv}\right)}^{0.02}=36$	$\eta /{\left(B_{iv}\right)}^{0.02}=34$	767.94	2029.64
Soil–XG 90d	$\eta /{\left(B_{iv}\right)}^{0.02}=36$	$\eta /{\left(B_{iv}\right)}^{0.02}=34$	1099.88	2882.24
Soil–XG–PPF 28d	$\eta /{\left(B_{iv}\right)}^{0.03}=36$	$\eta /{\left(B_{iv}\right)}^{0.03}=34$	874.79	2251.18
Soil–XG–PPF 90d	$\eta /{\left(B_{iv}\right)}^{0.03}=36$	$\eta /{\left(B_{iv}\right)}^{0.03}=34$	1201.24	3145.23

## Data Availability

Data are contained within the article.
